# Fat-free mass is associated with neurodevelopment outcomes in extremely preterm infants up to 3 years of age

**DOI:** 10.1038/s41390-025-04557-1

**Published:** 2025-11-11

**Authors:** Christoph Binder, Elisabeth Calek, Alexandra Thajer, Karin Harreiter, Nicholas Longford, Renate Fuiko, Angelika Berger, Katharina Goeral

**Affiliations:** 1 Comprehensive Center for Pediatrics, Department of Pediatrics and Adolescent Medicine, Division of Neonatology, Intensive Care and Neuropediatrics, Medical University of Vienna, Vienna, Austria; 2 Mohn Centre for Children’s Health and Wellbeing, School of Public Health, Imperial College London, London, UK

## Abstract

**Background:**

Extremely preterm infants represent a highly vulnerable population, facing a high risk of growth faltering and adverse neurodevelopment. Despite advances in neonatal care, nutritional strategies to support adequate growth, which is linked to improved neurodevelopment, remain uncertain. Body composition analysis, particularly fat-free mass (FFM), has emerged as a promising tool for evaluating nutritional status, and has been linked to larger brain size. However, its relationship with long-term neurodevelopment, especially in extremely preterm infants, remains unclear.

**Methods:**

The objective of this study was to investigate the association between early body composition changes, growth faltering, and neurodevelopmental outcomes, in a cohort of extremely preterm infants. This study included 105 infants born <28 weeks of gestation between 2017 and 2021, all of whom underwent body composition assessment at term-equivalent age and neurodevelopmental follow-up assessments at 1, 2, and 3 years of age.

**Results:**

FFM Z-Score was significantly and independently associated with higher neurodevelopmental scores up to 3 years of age. In contrast, fat mass Z-Score and growth faltering were not significantly associated with improved neurodevelopmental outcomes through 3 years of age.

**Conclusion:**

These findings suggest that early body composition analysis, particularly FFM, may serve as a potential predictor of long-term neurodevelopment.

**Impact:**

- Measurement of body composition, particularly fat-free mass (FFM), at term-equivalent age is associated with improved neurodevelopmental outcomes in extremely preterm infants.
- This study represents the largest cohort of extremely preterm infants in which FFM Z-Score rather than fat mass Z-Score, was positively associated with neurodevelopmental outcomes at 1, 2, and 3 years of age.
- Early gains in FFM appear to be critical for promoting favorable long-term neurodevelopmental outcome, underscoring the importance of targeted nutritional strategies.

## Introduction

Extremely preterm birth, defined as delivery before 28 weeks of gestation, remains a major global health concern due to its substantial contribution to neonatal morbidity and mortality.^1^ Survival rates have improved, and neonatal morbidities decreased over the last decades, due to advances in prenatal and neonatal care in this vulnerable population.^1^ Despite these improvements, extremely preterm infants remain at the highest risk for neonatal complications, and particularly impaired neurodevelopment.^2^ The underlying causes are multifactorial and diminish with increasing gestational age at birth.^3^ Optimal prenatal and neonatal management plays an essential role in reducing the risk of long-term neurological impairments in extremely preterm infants.^4^ Innovative and cutting-edge technologies, alongside with new pharmacological, and clinical interventions have contributed to a reduction of cerebral injuries.^5^ Furthermore, one of the most pressing concerns remains the impact of early nutrition on long-term neurological outcome.^6,7^ Several studies have emphasized that an early and adequate nutritional management is crucial for normal brain development.^8–10^ It is well known that extremely preterm infants are particularly susceptible to nutritional deficits and malnutrition during the neonatal period, when brain growth is most rapid and vulnerable to disruption.^11^ Human milk provides crucial nutrients and bioactive factors, and maternal milk feeding has been associated with improved neurodevelopment outcomes in preterm infants.^12,13^ However, despite its recognized importance, optimal nutritional strategies for this population remain poorly defined, and reliable parameters for assessing nutritional adequacy are still lacking.^14,15^

Technological advances in body composition assessment, such as air displacement plethysmography, have provided researchers and clinicians with more accurate tools to evaluate fat-free mass (FFM) and fat mass (FM), especially in preterm infants.^16^ These parameters are essential elements to evaluate the nutritional status and its relationship to organ development, including the brain.^17^ Our research group has previously shown that increased FFM at term-equivalent age significantly correlates with improved brain structural growth in extremely preterm infants.^18^ However, the optimal nutritional protocols that effectively promote FFM accretion without excessive fat gain remain undefined.^16^ Furthermore, the extent to which early FFM gains translate into long-term neurodevelopmental advantages has not been conclusively established, particularly in extremely preterm infants. It is also unclear whether these associations are consistent across different domains of neurodevelopment, such as cognitive, language, and motor function. Therefore, the primary objective of this study was to investigate the impact of body composition at term-equivalent age on long-term neurodevelopmental outcome up to 3 years of age in extremely preterm infants.

## Methods

This is a retrospective analysis of preterm infants, born at <28 weeks of gestation, who were previously included in a study investigating the association between body composition and brain size at term-equivalent age.^18^ The earlier study demonstrated a significant positive association between FFM and brain size.^18^ In the current study, we investigate the association between body composition (FFM Z-Scores and FM Z-Scores) at term-equivalent age and neurodevelopmental outcome at 1, 2, and 3 years of age. Study participants were born at the Medical University Hospital of Vienna, Austria (Department of Pediatrics and Adolescent Medicine; Division of Neonatology, Pediatric Intensive Care Medicine and Neuropediatrics) between November 2017 and March 2021. Exclusion criteria were intraventricular hemorrhage (IVH) > II, major congenital and chromosomal abnormalities, brain malformations, and metabolic disorders. The local Ethics Committee granted Ethical approval (Number: 2455/2020).

### Body composition

Body composition was assessed using air displacement plethysmography (Pea Pod, COSMED, Concord, California), a non-invasive and radiation-free technique.^19^ The Pea Pod is based on a two-compartment model of body composition, evaluating FFM and fat mass (FM). The accuracy and method of the device have been previously described in detail.^20^ It has been widely used to analyze body composition on preterm and term infants. The test duration is approximately seven minutes. The body composition measurement is part of a routine assessment at term-equivalent age (between 37 and 43 weeks postmenstrual age) for all infants born below 28 weeks of gestation in our institution. Due to procedural limitation, body composition measurement was only conducted in infants who were clinically stable and not receiving any respiratory support at the time of assessment. FFM and FM Z-Scores were derived using sex- and gestational-age-specific reference charts for preterm infants.^21^ Basic anthropometric measurements (weight, length, and head circumference) were analyzed concurrently with body composition measurements. Growth faltering was defined as changes in weight, length, and head circumference Z-Scores between birth and term-equivalent age.

### Neurodevelopmental outcome

Long-term neurodevelopmental outcomes were assessed using Bayley Scales of Infant Development third edition (Bayley-III) at 1, 2, and 3 years of age in our follow-up clinics by pediatricians and developmental psychologists using published German norms.^22^ Bayley-III measurements are routinely conducted for all infants born before 32 weeks of gestation in our institution. Developmental delays were classified according to conventional SD cut-offs as described by Johnson and Marlow^23^: scores ≥85 indicate normal development, 84–70 mild impairment, 69–55 moderate impairment, and <55 severe impairment. The test assesses cognitive, language, and motor domains.

### Anthropometric parameters and neonatal morbidities

Anthropometry (weight, length, and head circumference) was measured at birth, discharge, and at the timepoints of body composition and Bayley-III measurements. During the hospital stay, weight was assessed daily or every 48 h once an infant reached a body weight of 1000 g. Length panel was used to measure body length and flexible ruler for head circumference. Weight growth velocity, defined as the change in weight (gram/kg/day) from day 7 to day 28, was calculated. Changes in weight, length, and head circumference Z-Scores from birth until term-equivalent age were analyzed. Anthropometric data and Z-Scores were assessed using Fenton growth charts (up to 42 weeks of postmenstrual age),^24^ and WHO growth charts beyond 42 weeks of postmenstrual age.^25^ Neonatal morbidity was analyzed for retinopathy of prematurity (ROP) grade >II,^26^ intraventricular hemorrhage (IVH) > II,^27^ necrotizing enterocolitis (NEC) grade ≥II,^28^ bronchopulmonary dysplasia (BDP),^29^ and neonatal infection, defined as culture-proven septicemia with clinical signs of infection and antibiotic therapy for a minimum of 5 consecutive days. Small for gestational age was defined as a birth weight below the 10^th^ percentile for gestational age and sex.

### Nutritional management

The nutritional management was assessed in all infants from birth until discharge. The enteral and parenteral nutrient supply was followed according to ESPGHAN guidelines.^12,30^ Enteral nutrition was implemented on day one of life. Mother´s own milk is the gold standard in neonatal nutrition; when unavailable, pasteurized donor milk from a single donor was provided. Donor human milk was replaced with preterm formula milk at the postmenstrual age of 34 weeks of gestation. Preterm formula milk was switched to a standard term formula for infants with weight >10^th^ percentile or a nutrient-enriched post discharge formula for those <10^th^ percentile at term-equivalent age or upon hospital discharge. Feeding volumes were advanced daily by 20–30 ml per kilogram. Feeding intolerance was defined as gastric residuals exceeding 50% of the previous feeding volume, signs of abdominal distention or visible bowl loops, bloody stole, and/or severe vomiting. According to this definition, feeding advancement was reduced or withheld at the clinician´s discretion. Human milk supplementation was initiated when the infant reached 100 ml per kilogram per day using FM 85, Nestle, Vevey, Switzerland (in infants with a gestational age greater than 26 weeks) or Humavant +6, Prolacta Bioscience, California, United States of America (in infants with a gestational age below <26 weeks). Parenteral nutrition was started directly after birth and was discontinued once enteral intake reached 140–160 ml per kilogram per day. The amount and type of enteral feeds (exclusively human milk, standard term formula providing ~67 kcal per 100 ml, and nutrient-enriched post discharge formula providing ~75 kcal per 100 ml) were assessed from birth to discharge. The percentage of infants with maternal milk exposure at one year of age was evaluated.

### Statistical analysis

The data were analyzed using Statistical Package for the Social Sciences (SPSS) version 29 (IBM, New York, NY) and R version 4.2.1. (R Foundation for Statistical Computing, Vienna, Austria), with a significance level set at *p* < 0.05. Patient characteristics and anthropometric parameters, including body composition (FM and FFM), were presented as median and interquartile range (IQR) or as number and percentage (%), as appropriate. Anthropometric data and FFM as well as FM Z-Scores were standardized using growth charts adjusted for age and sex. Pearson correlation test was used to test the hypothesis of correlation between body composition (FFM and FM Z-Scores) and changes in weight, length, and head circumference Z-Scores from birth until term-equivalent age and neurodevelopmental outcome (Bayley Scales for cognitive, language, and motor domains) at 1, 2, and 3 years of age. Linear regression model was used to analyze the association between the body composition (FFM and FM Z-Scores) and changes in weight, length, and head circumference Z-Scores from birth until term-equivalent age, and Bayley Scales (cognitive, language, and motor domains) at 1, 2, and 3 years of age with adjustment for the following covariates, gestational age at birth and birth weight Z-Score,^31^ sex,^32^ length of parenteral nutrition,^33^ and the illness severity score (SNAPPE-II).^34^ Mean differences and adjusted mean differences were estimated and presented with 95% confidence intervals (CI).

## Results

During the study period, 139 infants were screened, and 105 infants (76%) were included in the analysis. Eleven infants (8%) were excluded due to intraventricular hemorrhage (IVH) greater than grade II. Body composition assessment at term-equivalent age was not performed in 21 infants (15%), due to the following reasons: continuous oxygen requirement: *n* = 3; hemodynamic instability: *n* = 2, device error n = 2, and parental refusal n = 14. Two other infants (1.4%) were excluded from the analysis due to loss to long-term follow-up.

Baseline characteristics and neonatal morbidities of the included patients are presented in Table 1. The median gestational age at birth was 26.1 weeks (IQR: 24.5, 27.0), and median birthweight 770 g (IQR: 648, 915). The median age at discharge was 38.0 weeks (IQR: 37.4, 39.4) and median weight at discharge 2960 g (IQR: 2530, 3570). The median SNAPPE-II score was 9 (IQR: 0, 9), indicating that most infants were classified as being at low risk for early mortality and morbidity among survivors. A majority of infants received an exclusively human milk diet (72%), and the median days on parenteral nutrition were 22 (IQR: 16, 36) (Table 1). Maternal milk exposure was observed in 38% (*n* = 40/105) of infants at one year of age.

**Table 1:** Patient´s characteristics.

Patient group	*n* = 105
Antenatal steroids for lung maturation	83 (79.0)
Infection-related delivery	61 (58.1)
PPROM	46 (43.8)
Cesarean section	90 (85.7)
Small for gestational age	9 (8.6)
Sex, Female	43 (41.0)
Umbilical artery pH, (median, IQR)	7.34 (7.29, 7.36)
Apgar Score, 5 min (median, IQR)	9 (8, 9)
Apgar Score, 10 min (median, IQR)	9 (9, 9)
SNAP-II Score, median (IQR)	9 (0, 9)
Anthropometry at birth	
Gestational age, week (median, IQR)	26.1 (24.5, 27.0)
Weight, gram (median, IQR)	770 (648, 915)
Weight, Z-Score (mean, 95%, CI)	0.1 (−0.6, 0.5)
Length, cm (median, IQR)	33.0 (31.0, 35.0)
Length, Z-Score (mean, 95%, CI)	0.0 (−0.6, 0.6)
HC, cm (median, IQR)	23.5 (22.0, 25.0)
HC, Z-Score (mean, 95%, CI)	0.0 (−0.5, 0.7)
Anthropometry at discharge	
Postmenstrual age, week (median, IQR)	38.0 (37.4, 39.4)
Weight, gram (median, IQR)	2960 (2530, 3570)
Weight, Z-Score (mean, 95%, CI)	−0.9 (−1.5, −0.5)
Length, gram (media, IQR)	48.0 (47.0, 52.0)
Length, Z-Score (mean, 95%, CI)	−0.7 (−1.5, 0.0)
HC, gram (median, IQR)	33 (31, 35)
HC, Z-Score (mean, 95%, CI)	−0.7 (−1.3, −0.3)
Neonatal morbidities	
NEC grade ≥III	5 (4.8)
ROP grade >II	2 (1.9)
Culture proven septicemia	18 (17.1)
Bronchopulmonary dysplasia	4 (3.8)
Total days on any parenteral nutrition, (median, IQR)	22 (16, 36)
Exclusively human milk diet	72 (69)
Standard term formula	28 (27)
Post-discharge formula	5 (4.8)

Data presented in median and IQR (interquartile range), mean and 95% confidence intervals, numbers, and percentages.

*PPROM* Preterm premature rupture of the membrane, *HC* Head circumference, *NEC* Necrotizing enterocolitis, *ROP* Retinopathy of prematurity, *IVH* Intraventricular hemorrhages, *PVL* Periventricular leukomalacia.

Body composition measurements and Bayley Scales-III up to 3 years of age are summarized in Table 2. The median age at body composition measurement was 41.2 weeks (IQR: 39.9, 44.2), and the median weight at measurement was 3396 g (IQR: 2907, 3970). The median FFM Z-Score was −1.5 (IQR: −2.5, −0.4) and median FM-Score 1.0 (IQR: 0.4, 1.7). Anthropometric Z-Score changes from birth until term-equivalent age are shown in Table 2. Most infants demonstrated normal development at 1 year of age (scores ≥85). At 2 and 3 years of age, the median scores on the Bayley Scales-III across the cognitive, language, and motor domains were below 85, indicating mild developmental impairment (Table 2). The median FFM- and FM Z-Scores and anthropometric Z-Score changes from birth until term-equivalent age in infants with Bayley-III scores <85 and ≥85 at 2 and 3 years of age are shown in Supplementary Table S1. Infants with Bayley-III scores <85 had consistently lower median Z-score in all outcome measures compared to those with scores ≥85 at 2 and 3 years of age. The largest difference was seen in FFM-Z-Scores, indicating a more substantial deviation in this parameter.

**Table 2:** Body composition, anthropometries, and Bayley Scale of Infants Development (Bayley-III).

Patient group	*n* = 105
Body composition and growth parameter	
Postmenstrual age, weeks (median, IQR)	41.2 (39.9, 44.2)
FFM, (%, IQR)	79.7 (75.1, 83.4)
FM, (%, IQR)	20.3 (16.6, 25.0)
FFM, gram (median, IQR)	2702 (2219, 3132)
FM, gram (median, IQR)	680 (501, 918)
FFM, Z-Score (mean, 95% CI)	−1.5 (−2.5, −0.4)
FM, Z-Score (mean, 95% CI)	1.0 (0.4, 1.7)
Weight, gram (median, IQR)	3396 (2907, 3970)
Weight, Z-Score (mean, 95% CI)	−1.0 (−1.6, −0.6)
Length, cm (median, IQR)	50.0 (48.0, 52.0)
Length, Z-Score (mean, 95% CI)	−1.3 (−2.2, −0.5)
HC, cm (median, IQR)	35 (33, 36)
HC, Z-Score (mean, 95% CI)	−0.6 (−2.2, −0.5)
Weight Z-Score changes, from birth to term-equivalent age	−0.9 (−1.4, −0.3)
Length Z-Score changes, from birth to term-equivalent age	−1.0 (−1.8, −0.6)
HC, Z-Score changes, from birth to term-equivalent age	−0.5 (−1.3, −1.0)
Bayley Scales at 1 year of age	
Cognition (median, IQR)	90 (80, 100)
Language (median, IQR)	100 (87, 106)
Motor (median, IQR)	92 (82, 103)
Weight, kg (median, IQR)	9.0 (8.5, 10.2)
Length, cm (median, IQR)	77.4 (75.6, 79.3)
HC, cm (median, IQR)	46.5 (44.0, 48.0)
Bayley Scales at 2 years of age	
Cognition (median, IQR)	80 (70, 99)
Language (median, IQR)	75 (57, 87)
Motor (median, IQR)	89 (79, 106)
Weight, kg (median, IQR)	12.8 (12.0, 13.8)
Length, cm (median, IQR)	88.0 (86.3, 90.1)
HC, cm (median, IQR)	48.5 (47.0, 49.5)
Bayley Scales at 3 years of age	
Cognition (median, IQR)	90 (75, 95)
Language (median, IQR)	81 (69, 91)
Motor (median, IQR)	79 (64, 85)
Weight, kg (median, IQR)	15.9 (14.7, 16.8)
Length, cm (median, IQR)	93.5 (92.1, 94.7)
HC, cm (median, IQR)	49.5 (48.5, 50.5)

Data presented in median and IQR (interquartile range), mean and 95% confidence intervals, and percentages (%).

*FFM* Fat-free mass, *FM* Fat mass, *HC* Head circumference.

**Fig. 1:**
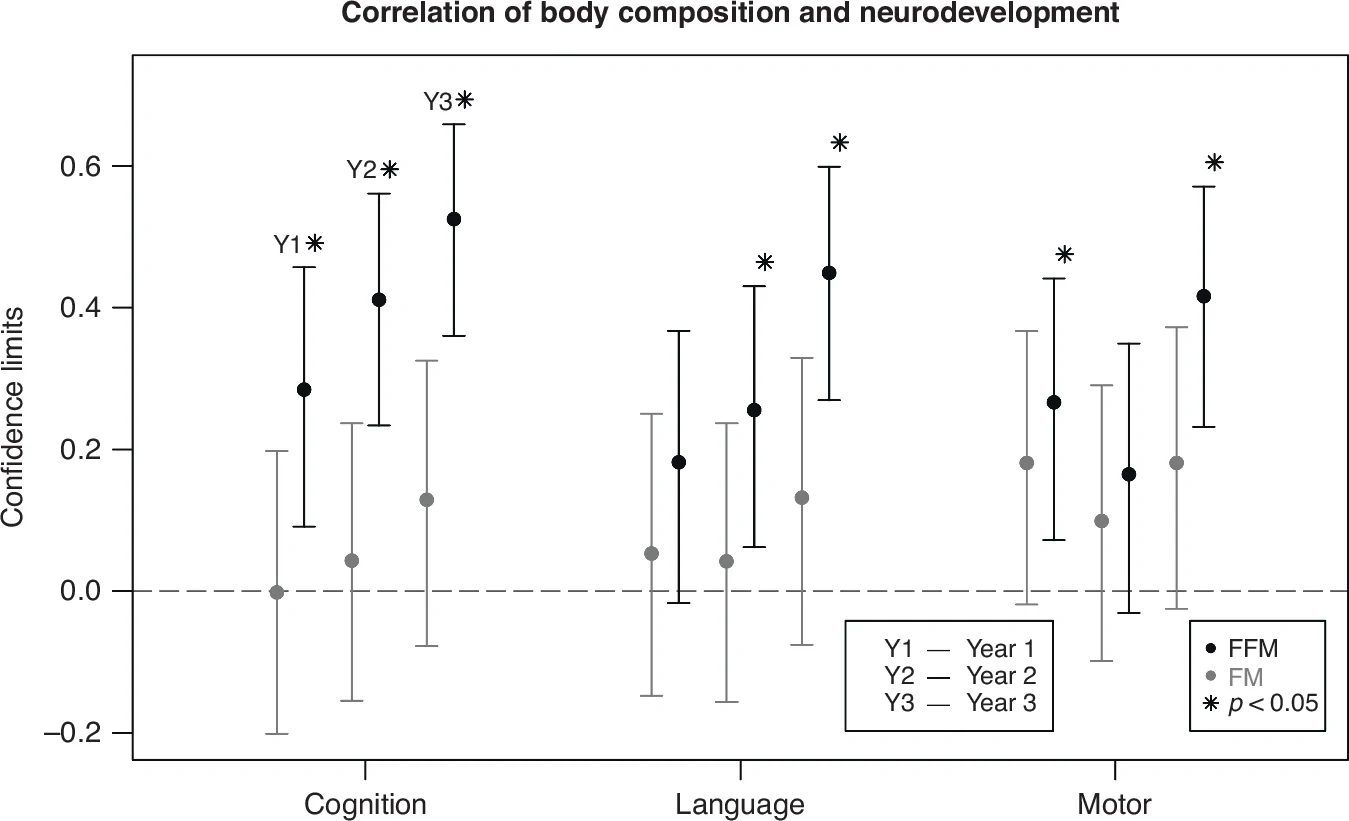
Correlation of body composition and neurodevelopment.

The correlation between body composition measurements and Bayley-III scores up to 3 years of age are represented in Table 3 and Fig. 1. Significant positive correlations were found between FFM Z-Scores and Bayley-III scores in all domains through 3 years of age. FM Z-Score showed no significant correlation with Bayley-III scores in any of the three domains at 1, 2, and 3 years of age (Table 3). Changes in weight Z-Score from birth until term-equivalent age were not significantly correlated with Bayley-III scores at any assessed time points, except for a significant correlation in the cognition and motor domains at 1 year of age. Changes in length Z-Score showed significantly positive correlation with cognitive and motor domains at 1, 2, and 3 years of age. No significant correlation was observed in the language domain up to 3 years of age. Changes in head circumference Z-Score were not significantly associated with Bayley-III scores, except for the motor domain at 1 year of age, and the cognitive and language domains at 3 years of age (Table 3).

**Table 3:** Correlation of body composition, anthropometric Z-Score changes from birth until term-equivalent age, and Bayley Scales of Infant Development up to 3 years of age.

	Bayley Scale (1 year)		Bayley Scale (2 years)		Bayley Scale (3 years)	
	Cognition	*p-value*	Cognition	*p-value*	Cognition	*p-value*
FFM, Z-Score	0.284 (0.091, 0.457)	*p* = *0.005*	0.411 (0.234, 0.561)	*p* < *0.001*	0.525 (0.360, 0.659)	*p* < *0.001*
FM, Z-Score	–0.002 (–0.201, 0.198)	*p* = *0.98*	0.043 (–0.155, 0.237)	*p* = *0.67*	0.129 (–0.078, 0.325)	*p* = *0.22*
Weight, Z-Score change	0.259 (0.032, 0.460)	*p* = *0.026*	0.169 (–0.056, 0.377)	*p* = *0.14*	0.268 (0.039, 0.470)	*p* = *0.023*
Length, Z-Score change	0.307 (0.008, 0.500)	*p* = *0.008*	0.318 (0.103, 0.505)	*p* = *0.005*	0.371 (0.153, 0.555)	*p* = *0.001*
HC, Z-Score change	0.071 (–0.160, 0.295)	*p* = *0.54*	0.173 (–0.112, 0.328)	*p* = *0.32*	0.319 (0.095, 0.513)	*p* = *0.006*
	**Language**		**Language**		**Language**	
FFM, Z-Score	0.182 (–0.017, 0.367)	*p* = *0.07*	0.255 (0.062, 0.430)	*p* = *0.010*	0.449 (0.269, 0.599)	*p* < *0.001*
FM, Z-Score	0.053 (–0.148, 0.250)	*p* = *0.60*	0.042 (–0.157, 0.237)	*p* = *0.68*	0.132 (–0.076, 0.329)	*p* = *0.21*
Weight, Z-Score change	0.150 (–0.082, 0.366)	*p* = *0.20*	0.144 (–0.083, 0.356)	*p* = *0.21*	0.170 (–0.066, 0.388)	*p* = *0.16*
Length, Z-Score change	0.169 (–0.035, 0.406)	*p* = *0.10*	0.195 (–0.030, 0.401)	*p* = *0.89*	0.215 (0.005, 0.415)	*p* = *0.07*
HC, Z-Score change	–0.032 (–0.259, 0.197)	*p* = *0.78*	0.138 (–0.089, 0.351)	*p* = *0.14*	0.179 ((–0.057, 0.396)	*p* = *0.13*
	**Motor**		**Motor**		**Motor**	
FFM, Z-Score	0.266 (0.072, 0.441)	*p* = *0.008*	0.165 (0.099, 0.349)	*p* = *0.09*	0.416 (0.232, 0.571)	*p* < *0.001*
FM, Z-Score	0.181 (–0.019, 0.367)	*p* = *0.07*	0.099 (–0.31, 0.290)	*p* = *0.32*	0.181 (–0.025, 0.372)	*p* = *0.08*
Weight, Z-Score change	0.367 (0.152, 0.550)	*p* = *0.001*	0.227 (0.007, 0.428)	*p* = *0.05*	0.208 (–0.025, 0.419)	*p* = *0.08*
Length, Z-Score change	0.348 (0.130, 0.534)	*p* = *0.002*	0.352 (0.140, 0.533)	*p* = *0.002*	0.308 (0.082, 0.504)	*p* = *0.009*
HC, Z-Score change	0.115 (–0.116, 0.335)	*p* = *0.033*	0.279 (0.060, 0.472)	*p* = *0.013*	0.267 (0.037, 0.469)	*p* = *0.024*

*FFM* Fat-free mass, *FM* Fat mass, *HC* Head circumference.

Data presents an estimate of correlation (95% confidence intervals), and *p*-values.

A significant and independent positive relationship between FFM Z-Score and Bayley-III scores was revealed by linear regression at 1 year of age (cognitive: estimated median 4.3, 95% CI: 1.5, 7.2; *p* = 0.003, language: estimated median 2.2, 95% CI: 0.7, 4.2; *p* = 0.043, and motor: estimated median 3.5, 95% CI: 1.1, 5.8; *p* = 0.004 domains). FFM Z-Score was independently and significantly associated with higher cognitive (estimated median 6.1, 95% CI: 2.8, 9.4; *p* < 0.001) and language (estimated median 4.6, 95% CI: 0.9, 8.4; *p* = 0.026) domains at 2 years of age. FFM Z-Score was not significantly associated with the motor domain (estimated median 1.3, 95% CI: –1.1, 3.8; *p* = 0.28) at 2 years of age. At 3 years of age, FFM Z-Score was significantly and independently positively associated with all three neurodevelopmental domains (cognitive: estimated median 5.7, 95% CI: 3.4, 8.1; *p* < 0.001, language: estimated median 5.0, 95% CI: 2.6, 7.4; *p* < 0.001, and motor: estimated median 4.3, 95% CI: 2.1, 6.5; *p* < 0.001). In the linear regression model, a 1 standard deviation increase in FFM-Z-Score was associated with a 4.3-point increase in Bayley-III scores. The duration of parenteral nutrition had a significant negative effect on the motor domain at 2 years of age: estimated median –0.2, 95% CI: –0.3, –0.1; *p* = 0.016, language domain at 3 years of age: estimated median –0.2, 95% CI: –0.3, –0.1; *p* = 0.039, and motor domain at 3 years of age: –0.16, 95% CI: –0.3, –0.03; *p* = 0.017.

No significant association was observed between FM Z-score and Bayley-III scores across all domains through 3 years of age (*p* > 0.05). Across all domains, changes in weight, length, and head circumference Z-Scores from birth until term-equivalent age showed no significant or independent association with Bayley-III scores through 3 years of age (*p* > 0.05).

## Discussion

This study investigated the relationship between body composition analysis at term-equivalent age and neurodevelopmental outcome, as measured by the Bayley Scales of Infant and Toddler Development, Third Edition (Bayley-III) at 1, 2, and 3 years of age in a cohort of extremely preterm infants. FFM Z-Score was found to be significantly and independently associated with higher Bayley-III scores across the domains of cognitive, language, and motor development throughout the first 3 years of life. In contrast, FM Z-Score and changes in weight, length, and head circumference Z-Scores from birth until term were not significantly associated with neurodevelopmental outcome. Inpatient gains in FFM appear to be critical for promoting favorable long-term neurodevelopmental outcome, underscoring the importance of targeted nutritional strategies in this high-risk population.

Developing optimal nutritional strategies for extremely preterm infants remains a profound challenge in clinical practice.^15^ Several studies have highlighted that adequate nutritional management, particularly in this vulnerable population, is essential for normal brain development.^6,11^ It is well established that mother´s own milk is the optimal source of nutrition for preterm infants, reducing the incidence of neonatal complications and improving long-term health outcomes.^6,12,13^ Furthermore, higher protein intake has been associated with improved brain metrics up to 7 years of age.^7^ Wang et al.^33^ also demonstrated that early insufficient parenteral macronutrient and energy intakes below current recommendations are associated with poor weight gain. However, effective nutritional strategies to enhance body composition and support neurodevelopment remain under investigation. Current concepts emphasize early parenteral nutrition, high macronutrients and energy intake, early initiation of enteral nutrition, and individualized fortification tailored to metabolic needs.^12,15,30,35^ Importantly, the nutritional management during hospital stay and in critical circumstances offers a key window of opportunity for supporting optimal growth and especially neurodevelopment.^36,37^ Furthermore, routine assessment of the nutritional status appears to be important for guiding individualized nutritional intervention.^35^ Body composition measurements has proven to be a useful tool for evaluating the nutritional status and optimizing management.^37,38^ Nevertheless, reliable methods for assessing and predicting long-term neurodevelopmental outcome, particularly in extremely preterm infants, have not yet been established. Despite extensive research, the impact of postnatal growth faltering on long-term neurodevelopmental outcome remains unclear due to conflicting evidence.^39,40^ Several studies demonstrated that growth faltering, including head growth, is associated with adverse neurodevelopment, but is not a strong or reliable predictor for long-term neurodevelopmental outcome.^39–41^ Advanced assessments, including magnetic resonance imaging (MRI) and body composition analysis, provide more accurate and comprehensive insights into somatic and brain growth.^42^ Our previous study showed that body composition, particularly FFM, is a stronger predictor for brain growth than traditional anthropometric parameters, with FFM being significantly and independently associated with greater brain size.^18^ Consequently, we investigated the effect of the body composition, particularly FFM on long-term neurodevelopment. In the current follow-up study, we found that FFM at term-equivalent age, was also an independent factor of neurodevelopmental outcome scores across the domains of cognitive, language, and motor development at 1, 2, and 3 years of age. In contrast, growth faltering, particularly Z-Score changes in head circumferences from birth to term-equivalate age, was correlated with adverse neurodevelopment, but this correlation was no longer significant after adjusting for covariates. Changes in weight, length, and head circumference Z-Scores from birth to term-equivalent age were not significantly nor independently associated with neurodevelopmental outcomes through 3 years of age. These findings are consistent with previous studies, highlighting that growth faltering may not be a reliable predictor of long-term neurodevelopmental outcomes.^40,41^ Growth faltering depends on several factors, including the presence of underlying medical and clinical conditions or infections, the timing and severity of nutritional deficits, and the preterm infant´s genetic predisposition.^43^ Additionally, growth faltering does not consider the complex interplay of metabolic and environmental factors, particularly in this vulnerable group of infants that can affect neurodevelopment.^40^ Therefore, it is important to consider a broader range of clinical tools, such as body composition analysis, for a more comprehensive understanding of an infant’s nutritional status and especially for long-term neurodevelopment. In our study, FFM – but not FM – was significantly associated with improved neurodevelopment, particularly after adjusting for covariates. Interestingly, FFM was significantly associated across all three neurodevelopmental domains up to 3 years of age, underscoring its potential as a strong predictor of neurodevelopment. On the other hand, FM was not significantly associated with long-term neurodevelopment. These results are consistent with our previously published study, in which FM was not significantly associated with larger brain size.^18^ Prior studies also support these results; Bell et al. found no significant association between FM and brain metric^17^, and Ramel et al.^37^ reported that greater gains in FFM, rather than FM, were associated with improved neurodevelopment in a small cohort of 34 preterm infants. We hypothesize that FM in preterm infants may not be a reliable indicator of neurodevelopment, as it primarily reflects overall adiposity rather than targeted brain growth and development. Moreover, increased fat accumulation may have a negative long-term effect on blood pressure, lipid metabolism and adiposity.^39,44^ In our study, FM Z-Scores were within normal range (median: 1.0; IQR: 0.4, 1.7), suggesting that these infants may not be at higher risk for long-term cardiovascular diseases. Nevertheless, long-term follow-up data may be valuable for evaluating its potential impact on cardiovascular health and obesity in later life. Building on these findings, previous studies demonstrated that higher protein and energy intake is associated with greater FFM,^44–46^ and may contribute to improved long-term neurodevelopment.^47^ Additionally, several other crucial factors are influencing FFM accretion, including among others protein-energy ratio, timing and amount of protein and energy intake, early enteral and parenteral feeding, and nutrient intake during critical illness.^48^ One limitation of our study is the lack of data on nutritional intake, and we can only hypothesize that nutrient intake was adequate in infants with higher FFM. Additionally, as this is an observational study, causal relationship cannot be established.

As expected, a longer duration of parenteral nutrition was negatively associated with neurodevelopmental outcome. It is well established that prolonged parenteral nutrition may result in nutrient deficiencies, hepatic dysfunction, neonatal infections, and metabolic alterations.^49,50^ We hypothesize that such effect may have contributed to alteration in body composition and compromised neurodevelopment in our cohort. Nevertheless, randomized controlled trials (RCTs) are warranted to investigate the impact of specific nutritional strategies, including the timing, composition, and duration of parenteral nutrition on long-term neurodevelopmental outcome in extremely preterm infants.

A key strength of our study is its focus on an especially vulnerable patient population, with a median gestational age at birth of 26 weeks, providing valuable new insights into the nutritional challenges faced by this high-risk group of preterm infants. The nutritional management in these infants is particularly complex due to their immature physiology, increased metabolic demands, and limited nutrient reserves.^11^ Consequently, extremely preterm infants are at increased risk for postnatal growth failure and adverse neurodevelopmental outcome.^6,33^ Our findings may therefore have a substantial impact on the evaluation of nutritional management and underscore the importance of regular body composition measurements in this population. However, the existing literature remains limited, with only a few studies conducted with small sample sizes are available.^36,37^ Our findings are consistent with the study by Pfister et al.^51^ who reported a significant positive correlation between FFM and neurodevelopmental outcome at 2 years of age. Nevertheless, their study did not specifically focus on extremely preterm infants, highlighting the relevance and novelty of our cohort-based study. Furthermore, in some of these studies neurodevelopmental outcome was assessed using parent-reported questionnaires^52^ or visual evoked potentials (VEPs).^36^ In contrast, our study employed the Bayley Scales of Infant and Toddler Development, Third Edition (Bayley-III), a standardized and reliable tool for assessing neurodevelopment in preterm infants.^53^ Additionally, our cohort is relatively large, consisting of 105 extremely preterm infants with a high neurodevelopmental follow-up rate of 98% (105 out of 107 infants) up to 3 years of age. Furthermore, infants with intraventricular hemorrhage grad >II were excluded from the analysis to ensure a low-risk preterm cohort. It is noteworthy that although most infants in our cohort demonstrated scores within the normal range at 1 year of age, the median cognitive, language, and motor scores were lower at 2 and 3 years, indicating evidence of mild developmental impairments. Several factors may account for this decline. First, developmental assessments at 2 and 3 years are more demanding, and therefore, more sensitive in detecting subtle neurodevelopmental deficits that may not be apparent at 1 year. The increasing complexity of cognitive and language tasks, in particular, can unmask early vulnerabilities in extremely preterm infants. Second, environmental influences, including variability in post-discharge care, access to early intervention services, and family socioeconomic status, are known to strongly influence neurodevelopmental outcomes beyond infancy. These findings underscore the importance of longitudinal follow-up beyond infancy, as early assessments may overestimate developmental potential in this high-risk population. To the best of our knowledge, this represents the largest cohort of extremely preterm infants in which the relationship between body composition, growth faltering and neurodevelopmental outcome has been evaluated. Our findings support the hypothesis that routine body composition assessments are a reliable bedside tool to evaluate nutritional status and may help guide interventions aimed at improving long-term neurodevelopmental outcomes. Further studies, particularly RCTs, are necessary to evaluate the impact of nutritional interventions on body composition and long-term neurodevelopment.

## Conclusion

This study investigated the association between early changes in body composition and neurodevelopmental outcomes up to 3 years of age, assessed by the Bayley Scales-III, in extremely preterm infants. We found that a higher FFM Z-Score at term-equivalent age is a potential predictor of neurodevelopmental outcomes at 1, 2, and 3 years of age. In contrast, FM Z-Score and growth faltering were not significantly associated with neurodevelopmental outcomes. These findings underscore the importance of individualized nutritional strategies aimed at supporting FFM accretion and enhancing long-term neurodevelopment in this high-risk population.

## Supplementary information


Supplementary Table S1


## Data Availability

The datasets generated and analysed during the current study are available from the corresponding author on reasonable request.
