# Supplementary material for: Fat-free mass is associated with neurodevelopment outcomes in extremely preterm infants up to 3 years of age
Source: Pediatr Res. 2025 Nov 11;100(1):249–56. doi: 10.1038/s41390-025-04557-1 (PMC13524817; doi:10.1038/s41390-025-04557-1)
Supplement: Supplementary file 1 [file 41390_2025_4557_MOESM1_ESM.pdf]

Supplementary Table S1. Body composition and anthropometric Z-Score changes from birth until term equivalent age at 2 and 3 years of age (Bayley-III Scores <85 and Scores ≥85)

	Bayley Scale (2 years)		Bayley Scale (2 years)	
	**Cognition Score <85**	*%*	**Cognition Score ≥85**	*%*
**FFM, Z-Score**	–2.1 (–2.8, –0.9)	*61*	–0.94 (–1.59, –0.16)	39
**FM, Z-Score**	0.98 (0.41, 1.58)	*61*	0.77 (0.44, 1.79)	*39*
**Weight, Z-Score change**	–1.0 (–1.4, -0.4)	*63*	–0.8 (–1.3, –0.2)	*27*
**Length, Z-Score change**	–1.3 (–2.0, -0.7)	*63*	–0.9 (–1.2, –0.4)	*27*
**HC, Z-Score change**	–0.6 (–1.4, -0.1)	*63*	–0.4 (–0.9, 0.2)	*27*
	**Language Score <85**		**Language Score ≥85**	
**FFM, Z-Score**	–1.49 (–2.62, –0.34)	71	–1.38 (–2.23, –0.51)	29
**FM, Z-Score**	0.92 (0.44, 1.51)	*71*	1.34 (0.33, 1.86)	*29*
**Weight, Z-Score change**	–0.85 (–1.60, –0.48)	*68*	–0.8 (–1.1, –0.1)	*32*
**Length, Z-Score change**	–0.10 (–1.92, –0.60)	*68*	–0.9 (–1.5, –0.4)	*32*
**HC, Z-Score change**	–0.50 (–1.33, –0.18)	*68*	–0.6 (–1.2, 0.1)	*32*
	**Motor Score <85**		**Motor Score ≥85**	
**FFM, Z-Score**	–1.59 (–2.64, –0.23)	50	–1.36 (–2.21, –0.58)	50
**FM, Z-Score**	0.95 (0.43, 1.54)	*50*	1.03 (0.43, 1.84)	*50*
**Weight, Z-Score change**	–1.05 (–1.63, –0.58)	*51*	–0.70 (–1.08, –0.12)	*49*
**Length, Z-Score change**	–1.35 (–2.10, –0.67)	*51*	–0.9 (–1.5, –0.5)	*49*
**HC, Z-Score change**	–0.75 (–1.61, –0.18)	*51*	–0.4 (–0.9, 0.2)	*49*
	**Bayley Scale (3 years)**		**Bayley Scale (3 years)**	
	**Cognition Score <85**		**Cognition Score ≥85**	
**FFM, Z-Score**	–2.2 (–2.97, –1.13)	*51*	–0.97 (–1.97, –0.12)	*49*
**FM, Z-Score**	0.94 (0.44, 1.40)	*51*	1.16 (0.29, 1.83)	*49*
**Weight, Z-Score change**	–1.10 (–1.67, –0.62)	*47*	–0.6 (–1.0, –0.1)	*53*
**Length, Z-Score change**	–1.60 (2.10, –0.72)	*47*	–0.85 (–1.35, –0.40)	*53*
**HC, Z-Score change**	–0.65 (–1.50, –0.30)	*47*	–0.25 (–0.9, 0.2)	*53*
	**Language Score <85**		**Language Score ≥85**	
**FFM, Z-Score**	–1.84 (–2.67, –0.87)	*62*	–1.01 (–2.08, –0.18)	*38*
**FM, Z-Score**	0.91 (0.41, 1.48)	*62*	1.16 (0.46, 1.80)	*38*
**Weight, Z-Score change**	–0.95 (–1.60, –0.60)	*56*	–0.6 (–1.2, –0.1)	*44*
**Length, Z-Score change**	–1.10 (–2.02, –0.48)	*56*	–0.90 (–1.55, –0.55)	*44*
**HC, Z-Score change**	–0.50 (–1.42, –0.20)	*56*	–0.5 (–1.2, 0.1)	*44*
	**Motor Score <85**		**Motor Score ≥85**	
**FFM, Z-Score**	–1.59 (–2.62, –0.24)	*82*	–1.04 (–2.10, –0.52)	*18*
**FM, Z-Score**	0.94 (0.41, 1.69)	*82*	1.0 (0.61, 1.75)	*18*
**Weight, Z-Score change**	–0.80 (–1.33, –0.27)	*83*	–0.9 (–1.3, –0.5)	*17*
**Length, Z-Score change**	–0.95 (–1.80, –0.58)	*83*	–1.25 (–1.63, –0.10)	*17*
**HC, Z-Score change**	–0.50 (–1.22, –0.10)	*83*	–0.75 (–1.45, 0.15)	*17*

Data presented in median and IQR (interquartile range), and percentages (%). Fat-free mass (FFM), Fat mass (FM), Head circumference (HC)
